# Unusual Combination of Lateral Condyle Mass Fracture and Olecranon Fracture in a Child: A Case Report

**DOI:** 10.7759/cureus.44706

**Published:** 2023-09-05

**Authors:** Nicolas O Quintero Cabrera, Cristal J Castellanos Mendoza, Jorge L Rojas Lievano, Jose D Cardona Ortegón, Jaime A Pedraza Yepes

**Affiliations:** 1 Department of Orthopedics and Traumatology, University Hospital Fundación Santa Fe de Bogotá, Bogotá, COL; 2 Department of Radiology, University Hospital Fundación Santa Fé de Bogotá, Bogotá, COL

**Keywords:** case report, orthopedic surgery, pediatric elbow injuries, olecranon fracture, lateral condyle fracture

## Abstract

Fractures of the lateral condyle and olecranon are two of the most common elbow injuries in the pediatric age group. However, their simultaneous occurrence is rare. Proper understanding and management of these injuries are essential to prevent long-term complications. This case report presents a patient who suffered both fractures, with surgical intervention for the condyle and non-surgical management for the olecranon. A two-year-old female child was brought to the emergency department following a fall from monkey bars, landing on her outstretched left arm. Clinical examination showed a markedly swollen and tender elbow with a restricted range of motion. No neurovascular deficit was noted. Plain radiographs revealed a displaced fracture of the lateral condyle and an associated non-displaced olecranon fracture. Given the displacement of the lateral condyle fracture, surgical intervention was deemed necessary. The patient underwent open reduction and internal fixation (ORIF) of the lateral condyle using Kirschner wires. The olecranon fracture, being non-displaced, was managed conservatively with a posterior splint. The patient’s postoperative recovery was uneventful. The Kirschner wires were removed at six weeks of follow-up, and active mobilization was started. The patient achieved full range of motion at three months post-injury. At a one-year follow-up, she had no pain, restriction, or any deformity, and radiographs confirmed the complete union of both fractures. Simultaneous fractures of the lateral condyle and olecranon in children are rare. The mechanism of injury is complex and warrants a high index of suspicion for associated injuries. Surgical fixation of the lateral condyle and conservative management of the olecranon fracture can yield excellent outcomes.

## Introduction

Pediatric elbow fractures are common orthopedic injuries, often resulting from falls or direct trauma to the elbow region. Among the various types of elbow fractures, lateral condyle and olecranon fractures are frequently encountered. Lateral condyle mass (LCM) fractures comprise approximately 12-20% of distal humerus fractures and are the second most common injury around the elbow in children after supracondylar fractures [1]. Most LCM fractures occur as an isolated injury. In contrast, olecranon fractures comprise approximately 4% of isolated elbow injuries in children, with 20% of cases presenting concomitant elbow injuries [2]. 

LCM fractures predominantly occur in children aged four to 10 years [3], while olecranon fractures are more commonly observed in older children and adolescents. Typically, these fractures are treated individually, adhering to established protocols based on fracture classification and growth plate involvement. The concurrent occurrence of an LCM fracture and an olecranon fracture in a single patient is an exceptionally rare entity, warranting comprehensive assessment and management to ensure optimal clinical outcomes. In an epidemiological study analyzing 2,502 elbow fractures in children over a 15-year period, only eight cases (0.3%) of this specific combination were reported [4].

In this case report, we present the case of a two-year-old female patient who presented to our orthopedic emergency room with severe elbow pain, swelling, and limited range of motion following a fall from a piece of playground equipment. Initial clinical examination and radiographic imaging revealed an unexpected combination of an LCM fracture and an olecranon fracture. The management of this unique combination fracture required a comprehensive treatment strategy, considering the specific anatomical characteristics and the potential for growth plate involvement. Surgical intervention was deemed necessary to achieve anatomic reduction of the lateral condyle fracture, while the olecranon fracture was managed non-operatively.

By presenting this case report, we aim to emphasize the importance of recognizing and appropriately managing unusual combinations of fractures in pediatric patients, and we hope to contribute to the existing literature, enhance clinical awareness, and improve the understanding of the diagnosis and management of this unusual combination fracture pattern.

## Case presentation

**Figure 1:**
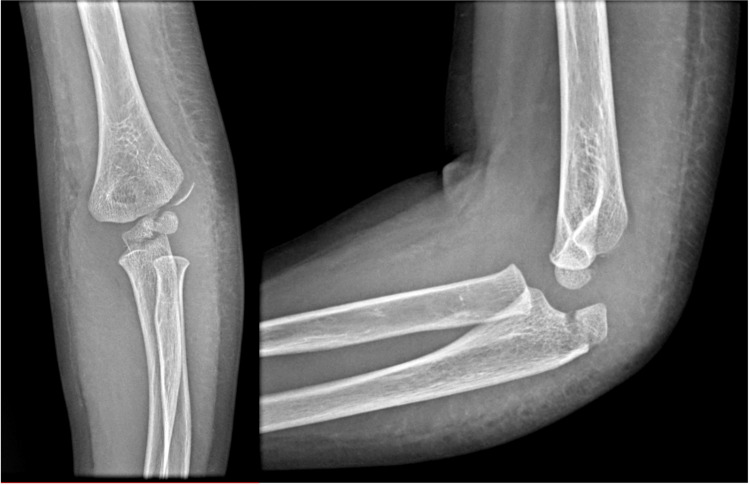
Preoperative radiographs (A) Anteroposterior and (B) lateral radiographs of the elbow, illustrating a displaced fracture of the lateral condyle and a nondisplaced fracture of the olecranon

A two-year-old female presented to the emergency department after a fall from monkey bars onto her outstretched left arm. She exhibited marked pain, swelling, and decreased elbow mobility. There was no history of prior elbow injuries or notable deformities. The clinical assessment highlighted moderate swelling with tenderness, primarily over the lateral and posterior elbow regions. No apparent deformity or compartment syndrome signs were observed, though the pain restricted motion, with no blockage to passive pronation-supination. Neurovascular status was intact. Radiographs identified a displaced LCM fracture, Weiss type III, accompanied by a non-displaced olecranon fracture (Figure 1).

Management 

**Figure 2:**
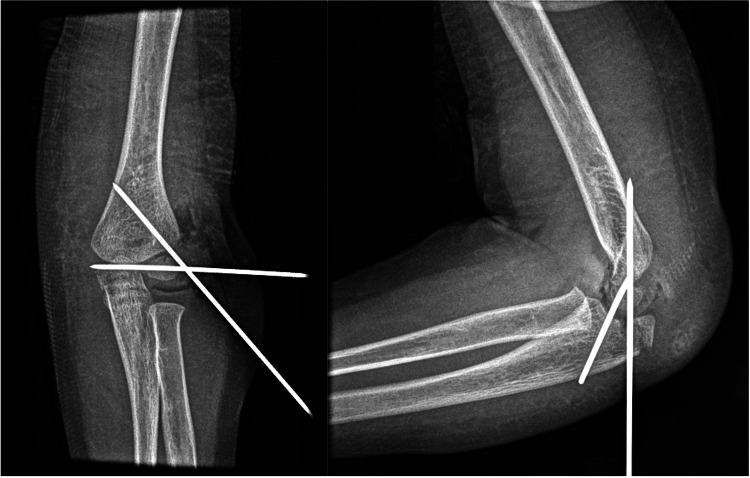
Immediate postoperative radiographs (A) Anteroposterior and (B) lateral radiographs of the elbow, revealing a successful reduction of the lateral condyle fracture and proper positioning of the Kirschner wires Importantly, the olecranon fracture sustained no displacement

The patient underwent surgical intervention for the LCM fracture under general anesthesia. The olecranon fracture did not require surgical management and was managed non-operatively. Open reduction and internal fixation (ORIF) were performed through a direct lateral approach using two smooth Kirschner wires under fluoroscopic guidance. After fixation of the LCM, the olecranon fracture was checked under fluoroscopy to confirm that there was no displacement or changes compared to preoperatively. There were no intraoperative or postoperative complications. Immediate postoperative radiographs confirmed an adequate reduction of the LCM fracture and position of the Kirschner wires (Figure 2). Postoperatively, the elbow was immobilized in a long posterior splint for six weeks to facilitate fracture healing and prevent displacement.

Postoperative course

**Figure 3:**
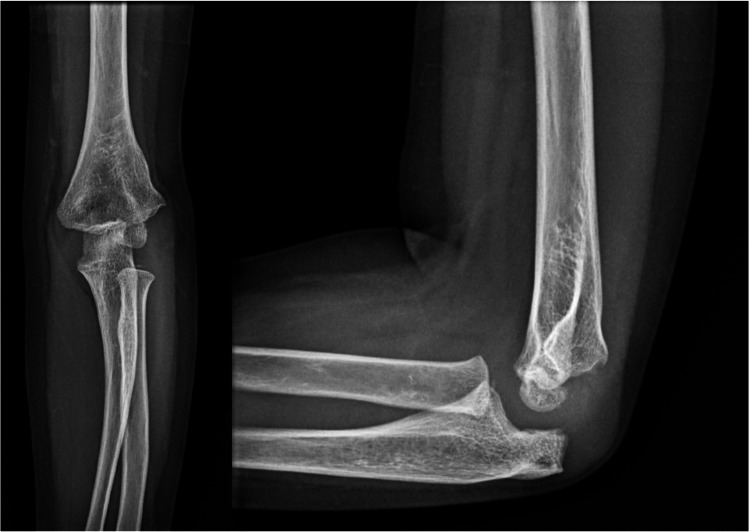
Six-week postoperative follow-up (A) Anteroposterior and (B) lateral radiographs of the elbow, demonstrating complete healing of both fractures with no evidence of malunion

**Figure 4:**
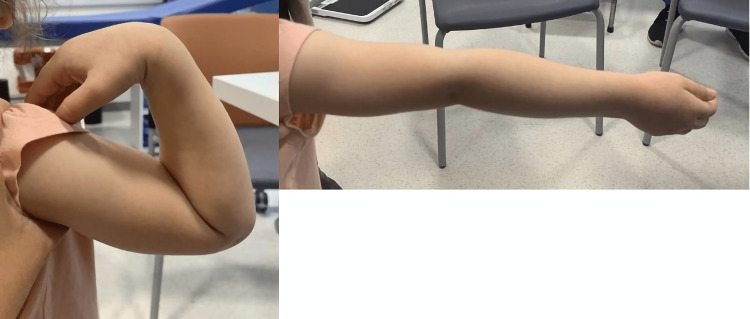
Three-month follow-up clinical assessment Clinical photographs illustrating the full range of motion in elbow flexion and extension, along with the absence of any deformities

Systematic follow-ups occurred at one week, four weeks, six weeks, three months, six months, and one year. At six weeks, the splint and wires were uneventfully removed. Radiographic assessments showed progressive healing of the LCM fracture with adequate alignment, and the olecranon fracture healed without displacement (Figure 3). By three months, the patient restored elbow function and resumed typical activities, with no functional deficits or growth disturbances (Figure 4).

## Discussion

In pediatric elbow injuries, concurrent LCM and olecranon fractures are seldom observed, making their management a point of clinical interest. This report details the experience of a two-year-old patient who sustained both injuries from a hyperextension event and underscores the importance of systematic treatment and follow-up.

LCM fractures are more common in children aged five to 10 years and often result from varus or valgus force between the lateral condyle and the radial head. Such fractures can sometimes be associated with other injuries like radial head or medial condyle fractures, especially after severe trauma [4]. Proper management of displaced LCM fractures is critical as they are categorized as Salter-Harris type IV injuries. Improper treatment can lead to complications like nonunion, avascular necrosis, deformities, and ulnar nerve injuries [5]. Weiss et al. [6] provided a classification for LCM fractures based on the displacement and rotation of the fracture fragment. Surgical treatment is recommended for displacements over 2 mm. Stage II fractures often involve closed reduction with percutaneous fixation, while stage III usually needs ORIF. A study of 20 LCM fractures associated with elbow dislocations or olecranon fractures showed that treatment decisions depended on the displacement of the condyle fracture [4]. Outcomes were predominantly favorable, though a minor subset did report limited motion, especially in extension.

The consensus on olecranon fracture treatment remains ambiguous. A review suggested non-operative treatment for displacements under 4 mm, reserving techniques like tension band fixation for displaced fractures [2]. Wilkins’ classification highlighted the importance of assessing the stability of the olecranon fracture under anesthesia before deciding on surgery [7]. A previous case report with a four-year-old [5] demonstrated successful use of Kirschner wires for treatment in treating a displaced LCM fracture combined with a displaced olecranon fracture, with good elbow mobility achieved at six months post-surgery. 

Lam and Mahadev [8] outline a treatment algorithm for cases that feature both LCM and olecranon fractures. The approach varies based on the displacement of each fracture. When both LCM and olecranon fractures are non-displaced, conservative management using immobilization and close monitoring is recommended. If the olecranon fracture is displaced but the LCM is not, surgical management with a posterior approach using tension wires for the olecranon is suggested. Concurrently, to mitigate potential displacement of the condyle, provisional fixation using percutaneous screws is advised. In situations where the LCM is displaced and the olecranon remains stable, ORIF of the condyle through a lateral approach are recommended. During this procedure, an intraoperative stability assessment of the olecranon is essential; if no displacement is detected, it remains unfixed. However, in cases where both fractures are displaced, a comprehensive ORIF for both injuries is conducted through a posterior approach, which includes an olecranon osteotomy.

Our concurrence with Lam and Mahadev’s proposed treatment algorithm is substantiated by our development of a comprehensive follow-up protocol for such complex elbow injuries. Irrespective of the specific scenario, we advocate for an initial follow-up assessment within the first and second week following hospital discharge. This evaluation serves the purpose of ascertaining the efficacy of immobilization and identifying potential immediate complications, such as infections, nerve injuries, or pressure-related issues. Additionally, an elbow X-ray aids in assessing fracture reduction and the positioning of osteosynthesis materials if surgical intervention is required. The second follow-up will take place roughly between the fourth and sixth weeks. At this stage, we expect to observe the first signs of radiological consolidation of the fracture. If appropriate, the immobilization and percutaneous nails can be removed, if they were used, allowing the start of physical therapy and spontaneous elbow mobilization. Moving forward, the three-month follow-up seeks to ensure complete fracture consolidation while assessing joint movement and identifying any stiffness, malunion, or deformities through clinical and radiological evaluation. At the six-month follow-up, our evaluation underscores the patient’s successful reintegration into daily activities, simultaneously addressing potential delayed complications such as malunion, cubitus valgus, late paralysis of the ulnar nerve, fishtail deformity, overgrowth or lateral prominence, and growth delay. Subsequent yearly assessments are tailored to individual patient requirements, guided by medical judgment, and underscore the significance of ruling out avascular necrosis.

**Figure 5:**
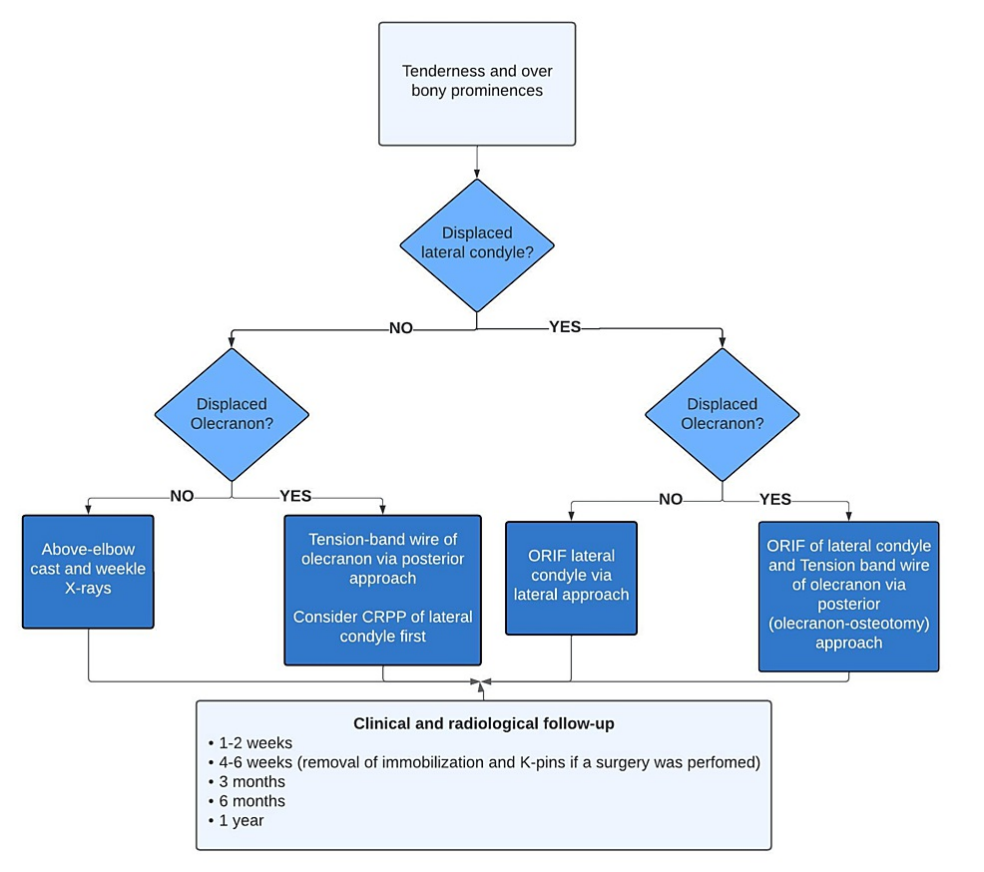
Algorithm for managing combined fractures of the LCM and olecranon LCM, lateral condyle mass; CRPP, closed reduction and percutaneous pinning; ORIF, open reduction and internal fixation Adapted from Lam et al. [8]

In this case, we effectively managed a displaced LCM fracture (Weiss type III) alongside a non-displaced olecranon fracture. Our approach, aligned with current evidence, involved ORIF using Kirschner wires for the LCM fracture. Simultaneously, the non-displaced olecranon fracture was treated conservatively. Over a year of follow-up, the patient experienced a successful recovery with complete elbow mobility, devoid of complications or deformities. This favorable outcome underscores the efficacy of our chosen therapeutic strategy. The results advocate for the adoption of a treatment and follow-up algorithm, offering a reliable roadmap for similar presentations in the emergency department (Figure 5).

## Conclusions

The rarity of concurrent LCM and olecranon fractures in pediatric patients underscores the importance of understanding optimal management strategies. This case report presents a comprehensive analysis of such a unique scenario, highlighting the successful application of evidence-based therapeutic approaches. The utilization of ORIF for the LCM fracture, combined with non-surgical management of the olecranon fracture, yielded excellent results. The absence of complications, restoration of full elbow mobility, and resumption of normal activities emphasize the effectiveness of our approach. The proposed treatment algorithm and meticulous follow-up protocol provide valuable guidance for similar cases, contributing to enhanced patient outcomes and clinical decision-making in the orthopedic emergency setting.
